# Real-time anatomy recognition in laparoscopic liver resection using video segmentation AI model

**DOI:** 10.1038/s41746-026-02989-5

**Published:** 2026-07-10

**Authors:** Haisu Tao, Kangwei Guo, Yijun Yang, Xincheng Yao, Ruiqiang Xiao, Xiaojun Zeng, Xuanshuang Tang, Lei Zhu, Yinling Qian, Jian Yang

**Affiliations:** 1 Department of Hepatobiliary Surgery I, General Surgery Center, Zhujiang Hospital, Southern Medical University, Guangzhou, China; 2 National Engineering Research Center of Innovation and Application of Minimally Invasive Instruments—South China Institute, Guangzhou, China; 3 Digital Intelligent Minimally Invasive Surgery Institute, Zhujiang Hospital, Southern Medical University, Guangzhou, China; 4 The Hong Kong University of Science and Technology (Guangzhou), Guangzhou, China; 5 Shenzhen Institute of Advanced Technology, Chinese Academy of Sciences, Shenzhen, China

**Keywords:** Computational biology and bioinformatics, Engineering, Mathematics and computing, Medical research

## Abstract

Laparoscopic liver resection (LLR) is challenging due to the complex and variable intrahepatic vascular anatomy, limited surgical field of view, and lack of tactile feedback, which collectively increase the risk of intraoperative injury. Accurate identification of anatomical structures is therefore essential for safe LLR. This study explores Vivim, a video segmentation model based on the Mamba architecture—a state-space model that integrates the long-range dependency modeling of transformers with the efficiency of recurrent networks. Evaluated on a multicenter dataset of 15,865 annotated frames from 45 videos, Vivim outperformed several image-based and video-based baselines, including U-Net, DeepLab v3 + , nnU-Net, FPN + EfficientNetV2-L, SegFormer, ConvLSTM-U-Net, ViViT and SegFormer + T-Mamba, in segmenting the Glissonean pedicle (GP) and hepatic vein (HV). By leveraging Mamba’s selective scanning and linear complexity, Vivim achieved Dice scores of 0.71 and 0.66 in single- and multi-target segmentation tasks, respectively, while maintaining real-time inference at 25 fps. The model demonstrated strong generalization on a cross-center external test set and robustness to typical surgical challenges. Clinically validated by 13 surgeons, Vivim improved recognition speed and accuracy, aiding intraoperative decision-making. Despite difficulties in differentiating similar vessels, this Mamba-based framework represents a promising step toward AI-assisted surgical navigation, bridging laboratory precision and clinical reliability.

## Introduction

Minimally invasive liver resection, encompassing robotic and laparoscopic approaches, has become the preferred surgical approach for patients with both benign and malignant liver diseases^1,2^. However, the complex and highly variable anatomy of intrahepatic vascular structures, combined with the limited surgical field of view and lack of tactile feedback in minimally invasive surgery, increases the risk of intraoperative injuries and complications^3–7^. Thus, accurate identification of key anatomical structures during surgery is essential for enabling safe surgical execution.

Recent advances in deep learning technologies have introduced novel methodologies and applications to computer-assisted surgery^8–14^. Numerous studies have investigated the potential applications of image-based models in surgical video analysis^15^. However, traditional image segmentation models face challenges when segmenting anatomical structures in surgical videos, often resulting in unclear boundaries and inconsistent masks across consecutive frames, revealing the limitations of single-frame image segmentation models^16^. This is primarily due to the inability of these models to effectively utilize the temporal information present in video data, leading to unstable segmentation results and insufficient recognition accuracy for small targets such as the liver’s vascular structures. Therefore, there is a need to delve into the temporal information of videos to enhance the accuracy and stability of anatomical recognition models.

Currently, mainstream video segmentation models predominantly utilize convolutional neural networks or Transformer architectures^17–19^. However, traditional Long Short-Term Memory (LSTM) frameworks struggle with processing long-duration videos^20^, while Transformer frameworks demand high computational resources^19,21^. In this context, the Mamba framework, based on state-space models, presents a promising solution^22^. Based on the Mamba framework, the Vivim model has demonstrated remarkable performance in ultrasound video segmentation tasks^23^. Mamba effectively captures the spatial-temporal information of video frames, improving segmentation accuracy and displaying notable advantages in long video understanding^23^, achieving a runtime that is six times faster than TimeSformer and reducing GPU memory requirements by 40% for processing 64-frame videos^24,25^, thus enabling real-time high-precision segmentation of high-resolution videos.

This study represents the first application of a video segmentation model for anatomical recognition in LLR. We develop an AI-driven anatomical recognition model based on video segmentation framework, and explore its value in identifying key structures during minimally invasive liver resection.

## Results

### Research design

In the first phase of this study, we trained several deep neural network models to predict the GP and HV regions on laparoscopic hepatectomy images, followed by comparative performance testing. Clinically oriented, we investigated two detection tasks: Task 1 (GP or HV) as a binary segmentation task distinguishing the unified foreground (either GP or HV) from the background, and Task 2 (GP + HV) as a multi-class segmentation task differentiating between GP, HV, and the background. Subsequently, the trained models were deployed for real-time inference on surgical video streams. In the second phase, we first invited multiple novice surgeons to observe intraoperative videos while the model performed real-time inference, comparing the recognition speed and accuracy between surgeons and the model. Second, a Likert-scale questionnaire was designed to quantitatively evaluate the perceived utility of the model, which was completed by both primary and senior surgeons after reviewing the real-time inference results.

### Model architecture

This study involves pixel-level semantic segmentation of the branches and main trunks of GP and HV exposed during liver resection surgery. We trained and compared seven models: U-Net^26^, DeepLab v3 + ^27^, nnU-Net^28^, FPN + EfficientNetV2-L^29,30^, SegFormer^31^, ConvLSTM-U-Net, ViViT^32^, SegFormer + T-Mamba and Vivim^23^.

U-Net, DeepLab v3 + , nnU-Net, FPN + EfficientNetV2-L, and SegFormer represent a spectrum of image-only architectures that emphasize spatial context as the primary signal for segmentation. U-Net presents a classic U-shaped structure with skip connections that enable precise localization by combining high-resolution and coarse features across multiple scales, underpinning robust performance even with limited data. DeepLab v3+ augments this by employing dilated convolutions to enlarge the receptive field without sacrificing boundary detail, thus capturing broader contextual cues such as surrounding tissue patterns. nnU-Net is a self-adaptive framework configuring itself to dataset characteristics including input size, patch sampling, and training schedules, offering practical benefits by controlling hyperparameter effects and reducing result variance across runs to ease cross-study comparability. FPN + EfficientNetV2-L combines a Feature Pyramid Network with a powerful backbone (EfficientNetV2-L) to effectively fuse multi-scale features, enabling robust performance across objects of varying size and texture while supporting stable segmentation under diverse intraoperative lighting and tissue variability. SegFormer replaces traditional CNN backbones with a lightweight vision transformer architecture, leveraging hierarchical self-attention for multi-scale feature extraction to capture both local details and global context, thereby enabling precise boundary delineation while maintaining computational efficiency for real-time surgical applications.

**Fig. 1:**
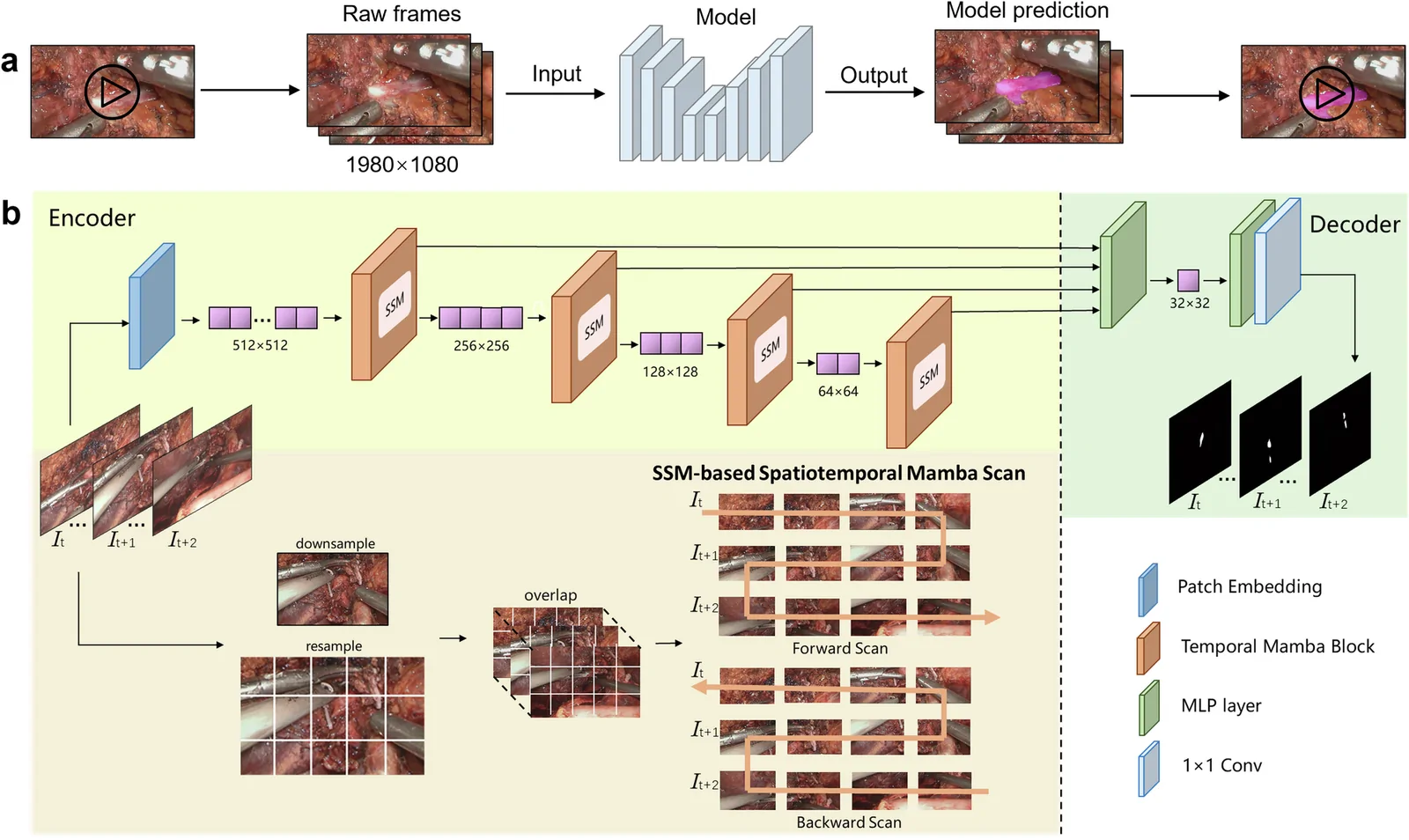
Details of network framework and model architecture. Panel **a** shows the network framework for live inference. Panel **b** shows the Vivim model architecture.

ConvLSTM-U-Net, ViViT, SegFormer + T-Mamba, and Vivim are included as video-based architectures. The ConvLSTM-U-Net incorporates convolutional LSTM for temporal modeling within the U-Net framework to capture dynamic information over short time intervals. ViViT applies transformer-based spatiotemporal attention to model long-range frame relationships for robust video segmentation by leveraging cross-frame dependencies to maintain consistency across dynamic scenes. SegFormer + T-Mamba integrates a bidirectional temporal module T-Mamba processing video sequences through forward and backward scans to leverage complementary temporal dependencies for maintaining consistent boundary evolution across dynamic surgical frames. Vivim is a video-based approach with a hierarchical encoder–decoder, where stacked temporal Mamba blocks enable spatiotemporal integration. Bidirectional temporal scanning and spatial sequences formed by temporally stacking frames enable comprehensive pixel information aggregation; a spatiotemporal selective scanning mechanism with parallel SSMs establishes long-range dependencies, and an auxiliary Sobel-based boundary head introduces boundary-aware constraints for improved delineation. The backbone utilizes SegFormer for high-resolution understanding, and inference proceeds without cropping, supporting real-time clinical video processing. To accommodate multi-label tasks, the loss function has been extended from binary segmentation to multi-class segmentation. The schematic diagram of the Vivim framework is shown in Fig. 1.

### Model performance on internal test set

In this study, we performed pixel-level semantic segmentation of the main trunks and branches of GP and HV exposed during laparoscopic liver resection. We trained and compared multiple models, including image-based and video-based models. All models underwent comprehensive hyperparameter tuning on a dedicated validation set. The optimal model for each network was selected by downloading the public implementation of each method and individually tuning its hyperparameters to identify the best-performing configuration, ensuring a fair comparison.

Vivim demonstrated superior performance in both single-target and multi-target segmentation tasks. For Task 1 (GP or HV), Vivim achieved a mean Dice score of 0.71, significantly outperforming all other models. Among the image-based baselines, U-Net, DeepLab v3 + , nnU-Net, FPN + EfficientNetV2-L, and SegFormer achieved mean Dice scores of 0.46, 0.47, 0.45, 0.58, and 0.64, respectively. The video-based baselines, ConvLSTM-U-Net, ViViT and SegFormer + T-Mamba, achieved scores of 0.50, 0.42, and 0.65, respectively (Table 1).

**Table 1:** Performance metrics for Task 1 (GP or HV) using image-based and video-based models on the internal test dataset

Model	Type	Param (M)	Dice Score (F1 Score)	mIoU	Accuracy	Precision	Recall	MAE	E-Measure	HD95	t-IoU	b-jitter	Inference speed (fps)
U-Net	Image	7.9	0.46 (0.29)	0.35 (0.25)	0.99 (0.01)	0.57 (0.33)	0.49 (0.34)	0.02	0.71 (0.19)	115.10 (9.62)	0.42 (0.10)	39.41 (6.59)	24
DeepLab v3+	Image	5.8	0.47 (0.17)	0.32 (0.14)	0.98 (0.01)	0.39 (0.19)	0.75 (0.27)	0.02	0.71 (0.18)	166.68 (9.93)	0.48 (0.09)	33.89 (3.83)	23
nnU-Net	Image	105.9	0.45 (0.25)	0.32 (0.21)	0.97 (0.02)	0.46 (0.28)	0.58 (0.31)	0.02	0.73 (0.18)	135.32 (9.62)	0.40 (0.09)	31.51 (4.22)	26
FPN + EfficientNetV2-L	Image	119.1	0.58 (0.27)	0.46 (0.25)	0.98 (0.02)	0.60 (0.29)	0.66 (0.29)	0.02	0.81 (0.16)	108.53 (9.67)	0.40 (0.06)	31.16 (4.09)	34
SegFormer	Image	47.3	0.64(0.27)	0.52(0.26)	0.98(0.01)	0.66(0.29)	0.71(0.28)	0.01	0.84(0.16)	87.38(9.46)	0.48 (0.12)	30.74 (4.05)	21
ConvLSTM-U-Net	Video	9.9	0.50 (0.25)	0.37 (0.22)	0.98 (0.01)	0.50 (0.28)	0.64 (0.30)	0.02	0.75 (0.18)	170.30 (10.41)	0.41 (0.10)	28.51 (3.86)	32
ViViT	Video	67.3	0.42 (0.26)	0.30 (0.21)	0.97 (0.07)	0.50 (0.29)	0.44 (0.30)	0.02	0.74 (0.15)	163.52 (10.52)	0.31 (0.10)	28.51 (3.86)	18
SegFormer + T-Mamba	Video	53.7	0.65 (0.25)	0.53 (0.25)	0.98 (0.02)	0.64 (0.28)	0.77 (0.25)	0.02	0.84 (0.18)	89.55 (9.38)	0.51 (0.11)	24.23 (2.95)	21
Vivim	Video	59.6	0.71 (0.25)	0.59 (0.25)	0.99 (0.01)	0.72 (0.27)	0.76 (0.26)	0.01	0.89 (0.14)	60.05 (7.75)	0.52 (0.12)	29.09 (3.25)	25

Data were expressed in mean (±standard deviation, SD). In this study, the Dice score and F1 score have the same values, and recall is also referred to as sensitivity. *mIoU* mean intersection over union, *MAE* mean absolute error, *t-IoU* inter-frame IoU, *b-jitter* boundary jitter, *fps* frame per second, *T-Mamba* temporal forward and backward SSM, *Vivim* Video Vision Mamba-based framework.

In the more challenging Task 2 (GP + HV), which requires simultaneous recognition of both structures, the performance of all models declined, yet Vivim maintained a clear lead with a mean Dice score of 0.66. The image-based models (U-Net, DeepLab v3 + , nnU-Net, FPN + EfficientNetV2-L, SegFormer) attained scores of 0.42, 0.44, 0.36, 0.37, and 0.61, respectively. The video-based baselines, ConvLSTM-U-Net, ViViT, and SegFormer + T-Mamba, achieved scores of 0.49, 0.37, and 0.62, respectively (Table 2). Vivim also consistently excelled in the boundary-sensitive metric HD95 across both tasks.

**Table 2:** Performance metrics for Task 2 (GP + HV) using image-based and video-based models on the internal test dataset

Model	Type	Target	Dice Score (F1 Score)	mIoU	Accuracy	Precision	Recall	MAE	E-Measure	HD95
U-Net	Image	GP + HV	0.42 (0.28)	0.31 (0.25)	0.99 (0.01)	0.76 (0.28)	0.35 (0.29)	0.02	0.64 (0.27)	92.20 (8.31)
DeepLab v3+	Image	GP + HV	0.44 (0.27)	0.32 (0.23)	0.99 (0.01)	0.67 (0.32)	0.40 (0.29)	0.02	0.70 (0.26)	100.75 (9.43)
nnU-Net	Image	GP + HV	0.36 (0.26)	0.26 (0.21)	0.97 (0.02)	0.52 (0.34)	0.34 (0.28)	0.02	0.74 (0.23)	124.94 (9.28)
FPN + EfficientNetV2-L	Image	GP + HV	0.37 (0.32)	0.27 (0.26)	0.98 (0.02)	0.52 (0.38)	0.36 (0.34)	0.02	0.64 (0.28)	97.93 (9.90)
SegFormer	Image	GP + HV	0.61 (0.26)	0.48 (0.25)	0.98 (0.02)	0.59 (0.29)	0.73 (0.26)	0.02	0.83 (0.19)	111.51 (9.88)
ConvLSTM-U-Net	Video	GP + HV	0.49 (0.29)	0.38 (0.25)	0.97 (0.02)	0.55 (0.33)	0.55 (0.32)	0.02	0.77 (0.23)	142.50 (11.19)
ViViT	Video	GP + HV	0.37 (0.26)	0.26 (0.21)	0.97 (0.02)	0.45 (0.31)	0.39 (0.30)	0.02	0.76 (0.22)	167.59 (11.19)
SegFormer + T-Mamba	Video	GP + HV	0.62 (0.26)	0.49 (0.25)	0.98 (0.02)	0.66 (0.28)	0.66 (0.28)	0.02	0.85 (0.18)	99.19 (9.86)
Vivim	Video	GP + HV	0.66 (0.26)	0.54 (0.26)	0.99 (0.01)	0.73 (0.27)	0.67 (0.29)	0.01	0.86 (0.17)	61.83 (7.86)

Data were expressed in mean (±standard deviation, SD). In this study, the Dice score and F1 score have the same values, and recall is also referred to as sensitivity. *mIoU* mean intersection over union, *MAE* mean absolute error.

**Fig. 2:**
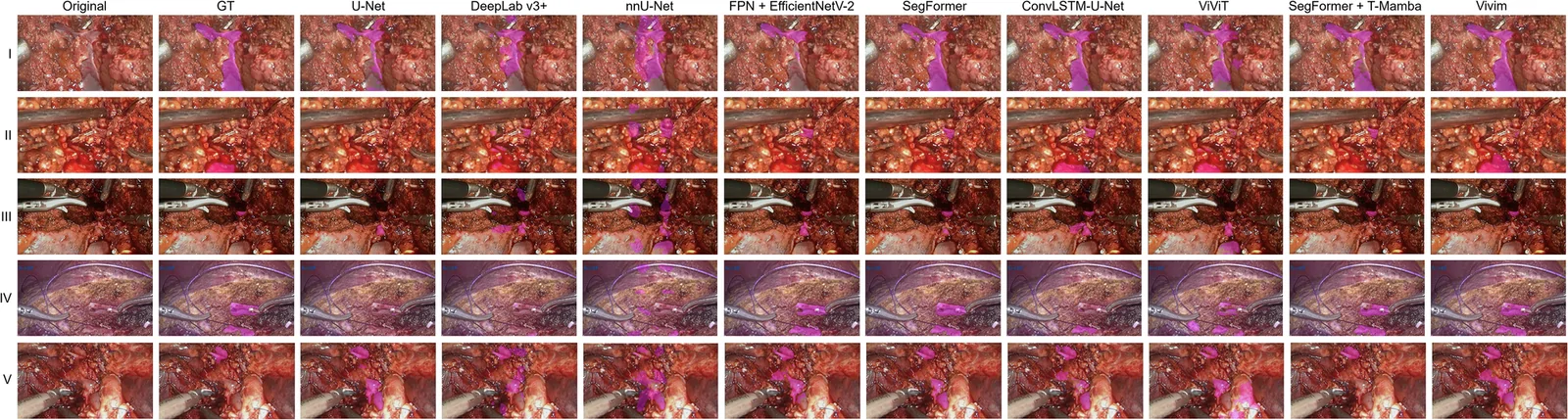
Examples of recognition performance of image-based and video-based methods under interference related to surgical operations during surgical procedures for Task 1 (GP or HV). **I** recognition of adequately exposed conduits in clean scenes; **II** bloodstained scenes; **III** target area too small for recognition; **IV** recognition of GP and HV simultaneously present; **V** misrecognition due to other tissues with similar visual characteristics. Original, original image; GT, ground truth.

**Fig. 3:**
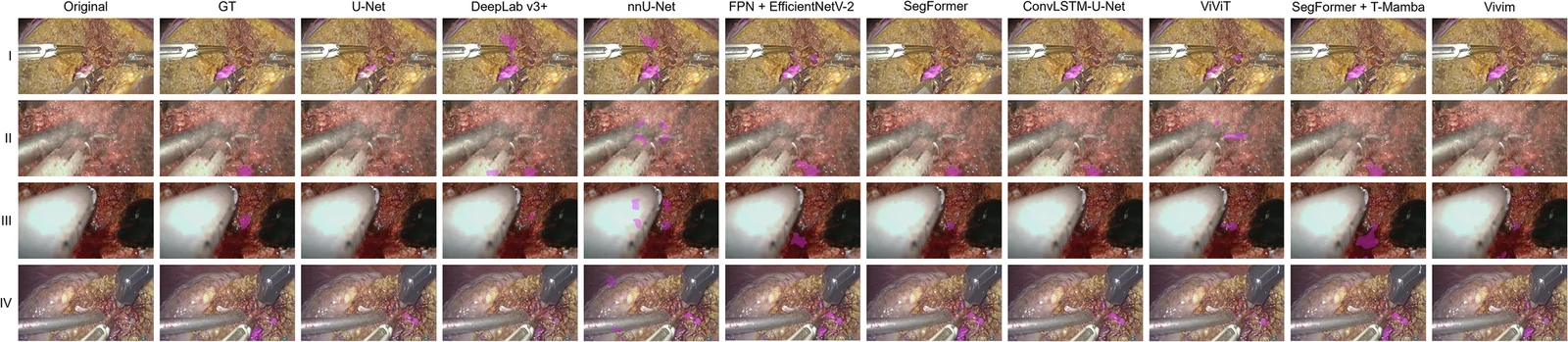
Examples of recognition performance of image-based and video-based methods under interference related to hardware devices during surgical procedures for Task 1 (GP or HV). **I** recognition of instrument occlusion of the target; **II** smoke obscuring the surgical field; **III** dim lighting caused by instrument occlusion; **IV** blurring and ghosting due to laparoscopic camera shake.

**Fig. 4:**
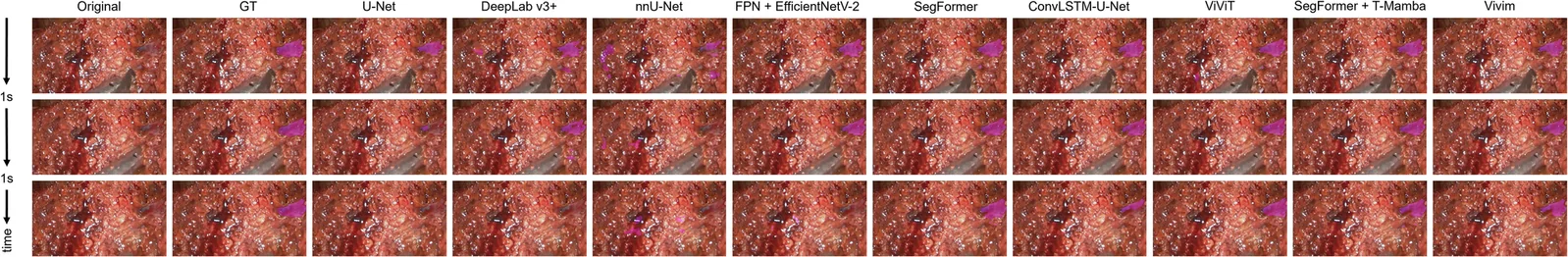
Visual comparison with image-based and video-based methods. The rows from top to bottom represent three consecutive video frames. All segmentation masks are overlaid in purple on the corresponding original frames. The video‑based methods exhibit superior temporal consistency across frames compared with image‑based methods.

**Fig. 5:**
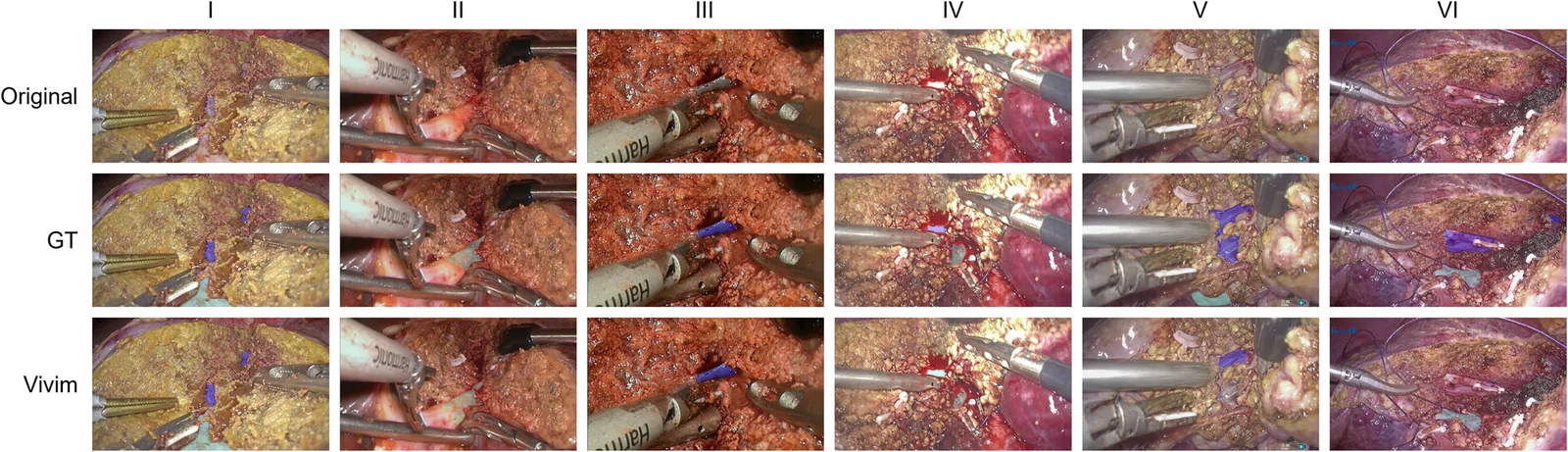
Examples of 2-class recognition performance of Vivim during surgical procedures for Task 2 (GP + HV) in an adequately exposed clean scene. **I** accurate recognition when both targets exist; **II** accurate recognition when GP exists independently; **III** accurate recognition when HV exists independently; **IV, V, VI** misrecognition arising from the similarity in visual characteristics of both targets when they are present.

To qualitatively illustrate Vivim’s robust segmentation capability, results were categorized and summarized across various surgical scenarios. Figure 2 displays segmentation outcomes for Task 1 under different surgical operation interferences, including motion artifacts, while Fig. 3 demonstrates performance under scenarios with hardware-induced interference. Furthermore, to analyze the impact of temporal integration, processing results across three consecutive frames sampled at 1-second intervals were visualized (Fig. 4). Exemplary recognition performance of Vivim on Task 2 is depicted in Fig. 5. To further evaluate the model’s ability to warn of potential failures, we conducted an uncertainty quantification study of Vivim on the internal test set for Task 1. The resulting estimates correlate moderately with segmentation errors (r = 0.44 pixel-wise) and are well-calibrated (ECE = 1.15%), indicating that Vivim can reliably trigger failure alerts in over 90% of critical cases (Supplementary Table 1).

Moreover, we evaluated Vivim’s temporal responsiveness by manually annotating 1861 abrupt appearance/disappearance events from the internal test set. Vivim achieved a Time-to-First-Correct-Mask (TTFM) of 0.22 s for partial disappearance and a detection lag of 2.07 s for abrupt appearance (Supplementary Table 2). No oversmoothing failures were observed, confirming that temporal integration does not cause harmful “ghosting”. However, 1503 under-smoothing cases indicate that maintaining prediction stability under visual ambiguity remains a challenge. Representative failure modes are illustrated in Supplementary Fig. 1, primarily due to insufficient spatiotemporal evidence accumulation and feature ambiguity.

Further, to assess the models’ performance across different dataset scenarios, two additional experiments were conducted. The first involved backbone recognition, where all models showed slight performance improvements; Vivim attained a maximum Dice score of 0.75, while DeepLab v3+ and U-Net scored 0.48 and 0.46 on average (Supplementary Table 3). The second experiment involved dataset cropping, where images were resized to 800 × 800, followed by training and testing. Model performance improved across all networks, with Vivim achieving an average Dice of 0.74, while DeepLab v3+ and U-Net scored 0.53 and 0.52, respectively (Supplementary Table 4).

Notably, Vivim processed high-resolution input frames at a rate of 25 fps, demonstrating its capability for real-time inference. This performance is illustrated in Supplementary Movie 1, which depicts intraoperative predictions for Task 1.

### Model performance on external test set

To rigorously evaluate the generalization ability of our model, testing was performed on a completely new and independent external validation set. This dataset was sourced from a distinct medical center and was not involved in any model training or hyperparameter tuning processes.

As shown in Tables 3 and 4, the Vivim model maintained leading and robust performance on this external test set, demonstrating effective generalization. For Task 1 (GP or HV), Vivim achieved a mean Dice score of 0.62, significantly outperforming the other compared models (U-Net: 0.38, DeepLab v3 + : 0.35, nnU-Net: 0.39, FPN + EfficientNetV2-L: 0.43, SegFormer: 0.58, ConvLSTM-U-Net: 0.46, ViViT: 0.29, SegFormer + T-Mamba: 0.59). Similarly, in the more complex Task 2 (GP + HV), Vivim sustained its advantage with a mean Dice score of 0.65, compared to the next best performer, SegFormer, which scored 0.59 (Table 4).

**Table 3:** Performance metrics for Task 1 (GP or HV) using image-based and video-based models on the external test dataset

Model	Type	Dice Score (F1 Score)	mIoU	Accuracy	Precision	Recall	MAE	E-Measure	HD95	t-IoU	b-jitter
U-Net	Image	0.38 (0.34)	0.29 (0.30)	0.99 (0.01)	0.45 (0.37)	0.40(0.39)	0.02	0.69 (0.21)	112.19 (10.14)	0.48 (0.15)	38.09 (13.65)
DeepLab v3+	Image	0.35 (0.23)	0.24 (0.17)	0.99 (0.01)	0.32 (0.23)	0.52(0.35)	0.03	0.71 (0.22)	173.21 (10.24)	0.54 (0.18)	33.57 (10.00)
nnU-Net	Image	0.39 (0.27)	0.28 (0.22)	0.98 (0.01)	0.37 (0.28)	0.52(0.35)	0.03	0.67 (0.12)	143.12 (9.62)	0.48 (0.21)	34.73 (12.00)
FPN + EfficientNetV2-L	Image	0.43 (0.32)	0.33 (0.27)	0.98 (0.01)	0.47 (0.32)	0.49(0.37)	0.01	0.75 (0.19)	142.27 (11.17)	0.41 (0.14)	29.09 (4.95)
SegFormer	Image	0.58 (0.26)	0.45 (0.25)	0.98 (0.01)	0.51 (0.27)	0.81 (0.26)	0.02	0.78 (0.19)	106.35 (10.45)	0.42 (0.15)	29.04 (3.22)
ConvLSTM-U-Net	Video	0.46 (0.26)	0.34 (0.22)	0.98 (0.01)	0.43(0.27)	0.62(0.33)	0.01	0.76 (0.17)	193.36 (10.56)	0.47 (0.13)	28.25 (4.68)
ViViT	Video	0.29 (0.28)	0.20 (0.22)	0.98 (0.02)	0.28(0.28)	0.37(0.36)	0.01	0.72 (0.15)	224.98 (11.87)	0.31 (0.11)	40.04 (6.81)
SegFormer + T-Mamba	Video	0.59 (0.26)	0.47 (0.25)	0.99 (0.02)	0.52 (0.27)	0.81 (0.25)	0.02	0.79 (0.18)	106.58 (10.90)	0.45 (0.17)	25.05 (3.26)
Vivim	Video	0.62 (0.29)	0.51 (0.28)	0.99 (0.01)	0.57 (0.30)	0.76 (0.32)	0.01	0.87 (0.18)	65.79 (8.91)	0.54 (0.16)	24.08 (3.97)

Data were expressed in mean (±standard deviation, SD). In this study, the Dice score and F1 score have the same values, and recall is also referred to as sensitivity. mIoU mean intersection over union, MAE mean absolute error, *t-IoU* inter-frame IoU, *b-jitter* boundary jitter.

**Table 4:** Performance metrics for Task 2 (GP + HV) using image-based and video-based models on the external test dataset

Model	Type	Target	Dice Score (F1 Score)	mIoU	Accuracy	Precision	Recall	MAE	E-Measure	HD95
U-Net	Image	GP + HV	0.30 (0.33)	0.23 (0.28)	0.99 (0.01)	0.48 (0.43)	0.26 (0.32)	0.01	0.55 (0.30)	78.69 (7.94)
DeepLab v3+	Image	GP + HV	0.32 (0.29)	0.23 (0.22)	0.99 (0.01)	0.47 (0.37)	0.31 (0.31)	0.01	0.62 (0.29)	97.96 (8.46)
nnU-Net	Image	GP + HV	0.33 (0.28)	0.23 (0.22)	0.98 (0.01)	0.45 (0.35)	0.32 (0.31)	0.01	0.71 (0.25)	125.45 (10.16)
FPN + EfficientNetV2-L	Image	GP + HV	0.27 (0.31)	0.20 (0.25)	0.98 (0.01)	0.37 (0.38)	0.28 (0.34)	0.01	0.57 (0.30)	91.23 (11.27)
SegFormer	Image	GP + HV	0.59 (0.27)	0.47 (0.26)	0.99 (0.01)	0.55 (0.28)	0.74 (0.30)	0.01	0.84 (0.18)	94.44 (10.08)
ConvLSTM-U-Net	Video	GP + HV	0.56 (0.33)	0.45 (0.29)	0.99 (0.01)	0.59 (0.33)	0.60 (0.36)	0.01	0.80 (0.25)	90.43 (10.51)
ViViT	Video	GP + HV	0.35 (0.29)	0.25 (0.23)	0.98 (0.01)	0.39 (0.31)	0.39 (0.34)	0.01	0.77 (0.22)	187.22 (12.02)
SegFormer + T-Mamba	Video	GP + HV	0.58 (0.27)	0.45 (0.29)	0.99 (0.01)	0.55 (0.29)	0.71 (0.30)	0.01	0.82 (0.20)	93.47 (9.79)
Vivim	Video	GP + HV	0.65 (0.27)	0.53 (0.27)	0.99 (0.01)	0.65 (0.29)	0.72 (0.29)	0.01	0.87(0.18)	66.01 (8.65)

Data were expressed in mean (±standard deviation, SD). In this study, the Dice score and F1 score have the same values, and recall is also referred to as sensitivity. *mIoU* mean intersection over union, *MAE* mean absolute error.

These results provide strong evidence that the performance gains demonstrated by Vivim are not attributable to overfitting on a specific dataset but rather reflect its powerful generalization capability, enabling effective adaptation to unseen data from different medical institutions. This finding offers crucial support for the potential broad application of Vivim in diverse real-world clinical environments.

### Evaluation on public benchmarks

To further assess Vivim’s generalization, we evaluated it on a public surgical segmentation dataset: the Dresden Surgical Anatomy Dataset (DSAD)^33^. By merging the “Inferior Mesenteric Artery” and “Intestinal Veins” classes into a unified “Vessel” category, we constructed a single-target segmentation task that reflects the complexity of hepatic vessel segmentation, resulting in a total of 2157 frames. As shown in Supplementary Table 5, Vivim consistently outperformed all compared state-of-the-art methods on the benchmark, providing strong independent evidence of its effectiveness and generalizability.

### Component-wise ablation study of Vivim

To dissect the contribution of each core component within the Vivim model, a systematic ablation study was conducted on the internal test set for Task 1, with the results summarized in Table 5.

**Table 5:** Ablation study on Vivim design

	ST-Mamba			Sobel boundary head	Dice Score (F1 Score)	mIoU	Accuracy	Precision	Recall	MAE	E-Measure	HD95
	T^f^	T^b^	S									
Basic (SegFormer)	–	–	–	–	0.64 (0.27)	0.52 (0.26)	0.98 (0.01)	0.66 (0.29)	0.71 (0.28)	0.01	0.84 (0.16)	87.38 (9.46)
C1 (Basic + T^f^)	√	–	–	–	0.65 (0.26)	0.53 (0.26)	0.98 (0.01)	0.62 (0.28)	0.80 (0.23)	0.03	0.81 (0.19)	92.10 (9.65)
C2 (Basic + T^f^ + T^b^)	√	√	–	–	0.65 (0.25)	0.53 (0.25)	0.98 (0.02)	0.64 (0.28)	0.77 (0.25)	0.02	0.84 (0.18)	89.55 (9.38)
C3 (Basic + T^f^ + T^b^ + S)	√	√	√	–	0.68 (0.24)	0.56 (0.24)	0.99 (0.01)	0.70 (0.26)	0.76 (0.25)	0.01	0.86 (0.15)	73.88 (8.43)
Vivim	√	√	√	√	0.71 (0.25)	0.59 (0.25)	0.99 (0.01)	0.72 (0.27)	0.76 (0.26)	0.01	0.89 (0.14)	60.05 (7.75)

Data were expressed in mean (±standard deviation, SD). In this study, the Dice score and F1 score have the same values, and recall is also referred to as sensitivity.

*T*^*f*^ temporal forward SSM, *T*^*b*^ temporal backward SSM, *S* spatial SSM, *mIoU* mean intersection over union, *MAE* mean absolute error.

We constructed four baseline networks from Vivim. The first baseline, denoted as “Basic,” removes all Mamba layers and boundary-aware constraints from Vivim, which corresponds to the SegFormer architecture. The second baseline (C1) incorporates ST-Mamba layers with a temporal forward SSM (T^f^) into the Basic framework. Subsequently, the third baseline (C2) further adds a temporal backward SSM (T^b^) to the ST-Mamba structure. The fourth baseline (C3) then integrates a spatial SSM (S) into the configuration of the third baseline. Finally, we augmented the model with the Sobel boundary head, resulting in the complete Vivim architecture.

The ablation study clearly demonstrates that: The Bidirectional Scanning mechanism contributes substantially to the performance gain by effectively aggregating contextual information from both past and future frames, thereby enhancing spatiotemporal feature representations. The Selective State Update, core to the Mamba architecture, provides crucial performance improvements by selectively retaining and propagating essential information vital for video segmentation. The Sobel boundary head shows distinct value in refining boundary metrics, particularly HD95, by explicitly guiding the model to focus on boundary regions, resulting in more anatomically precise segmentation, especially for small vessel structures.

In conclusion, the final superior performance of the Vivim model results from the synergistic effect of these designed components, each contributing significantly to different aspects of the model’s overall capability.

### Real-time inference

In this study, the deployed Vivim framework was effectively implemented during liver resection surgeries at Zhujiang Hospital. High-definition endoscopic video feeds (1920 × 1080 pixels) were transmitted from the camera control unit (CCU) to a laptop. This setup facilitated real-time anatomical structure detection. The processing was conducted on a laptop equipped with an Intel Core i7 processor, 16 GB RAM, and an NVIDIA GeForce RTX 4090 GPU. To achieve real-time inference while processing high-resolution laparoscopic videos, the model was deployed with a specific configuration. During inference, input frames with an original resolution of 1920 × 1080 pixels were resized to 512 × 512 pixels and normalized. The model processes input in the form of video clips. To optimize the trade-off between capturing sufficient temporal dynamics for videos and GPU memory constraints, a batch size of 2 clips (5 frames per clip) was selected to prevent out-of-memory errors while maintaining effective temporal modeling. Consequently, each forward pass processes a total of 10 frames (2 clips × 5 frames). During real-time inference, the trained model successfully identified the exposed GP and HV throughout the surgical procedure. It continuously captures the latest images from the laparoscopic stream, which are buffered in a system designed for sequential processing. The model predicts the buffered images at an average rate of 25 fps, corresponding to approximately 39.90 milliseconds per frame, thus enabling rapid real-time predictions. This high processing speed is critical for providing real-time guidance of key anatomical structures during surgery.

### Clinical performance evaluation

To evaluate whether the AI model Vivim can accurately recognize and visualize data from multiple perspectives, and to gather subjective qualitative assessments from surgeons, we designed an experiment based on our prior study. Six primary surgeons each viewed 20 independent surgical video clips for Task 1 and another 20 for Task 2 (none of which had been used for training or validation), while the trained model performed real-time inference, enabling a comparative analysis of recognition speed and accuracy between surgeons and the deep learning model (Supplementary Note 1 and Supplementary Table 6)^15^. The criterion for “first recognition” was rigorously defined: for the AI model inferencing at 25 fps, it was the first frame where a structure (GP or HV) was predicted with an IoU >0.5; for the surgeons, it was the frame where they first paused the video and annotated the region of interest. To ensure a fair comparison, the recognition moment for both was determined based on the video timestamp, thereby eliminating bias from varying surgeon reaction times. Two senior surgeons reviewed all annotations made by both the primary surgeons and the AI model, confirming that these annotations were not false positives (IoU <0.5). If the primary surgeons failed to pause in time upon initial recognition, the corresponding data were excluded from analysis. Given Vivim’s superior segmentation results, this model was chosen to evaluate recognition performance. The Vivim-first recognition rate for Task 1 (GP or HV) was 90.0%. For Task 2 (GP + HV), first recognition by the model and surgeons was 61.5% and 38.5%, respectively. Further per-structure analysis revealed that HV was consistently more readily identified than GP across both tasks, providing deeper insights into the model’s performance characteristics.

Furthermore, to assess whether the AI could recognize and visualize data from multiple perspectives and to obtain subjective quantitative evaluations from surgeons, we employed a Likert scale questionnaire comprising six criteria (Supplementary Table 7)^34^. For the scoring-based evaluation, 13 surgeons participated, divided into an expert group (7 board-certified senior surgeons) and a novice group (6 primary surgeons). Each surgeon watched four brief prediction videos and rated each item on the five-point Likert scale. Critically, each surgeon rated four videos that were generated from a test dataset completely separate from the training sets, mitigating potential bias from prior exposure. As shown in Supplementary Table 8, the average scores for all evaluation items ranged from 3 to 5. The highest scores were observed for Questions 4 and 5, which reflected surgeons’ impressions of the segmentation quality. Notably, the novice group provided significantly higher ratings compared to the expert group. The four short videos depicting AI-based recognition of GP and HV were generated from datasets different from the training sets.

## Discussion

This study addresses the challenge of identifying key anatomical structures during LLR, innovatively utilizing a Mamba-based video understanding framework for model training. This approach incorporates temporal information from video frames to perform semantic segmentation, enabling precise recognition of the GP and HV. Moreover, to enhance the generalizability of AI models, we established a multicenter cohort comprising heterogeneous clinical data from 4 institutions. Compared to traditional single-frame segmentation models and other advanced baselines including nnU-Net, FPN + EfficientNetV-2, SgeFormer, ConvLSTM-U-Net, ViViT and SegFormer + T-Mamba, Vivim demonstrates superior performance in both single-target and multi-target recognition tasks, particularly under complex surgical scenarios involving blood stains and smoke interference. During real-time inference, Vivim processes high-resolution videos at 25 fps, with an average latency of only 39.9 milliseconds per frame, satisfying clinical real-time demands. Clinical evaluation indicates that its recognition accuracy is comparable to that of novice surgeons and that it effectively highlights lesions that are otherwise prone to being overlooked, earning high approval from practicing surgeons. To our knowledge, this study is the first to technically validate intraoperative video segmentation methods for identifying anatomical structures during LLR.

For AI studies targeting clinical applications, selecting an appropriate model is of paramount importance. Several studies have explored the clinical potential of different AI models in LLR. For instance, Norikazu Une et al. evaluated the ability of an AI model (EfficientNetV2-L) to recognize vascular structures during LLR^30^. Additionally, our previous research investigated the performance of nnU-Net and DeepLab v3+ in vascular segmentation within LLR^15^, confirming the potential of these techniques to provide real-time guidance and reduce adverse events. However, we identified limitations in these existing image-based models, including unclear boundaries and inconsistent segmentation masks across consecutive video frames, which hinder their clinical application. To address these issues, this study introduces an innovative application of Vivim, a Mamba-based state-space video understanding framework, for real-time semantic segmentation of hepatic vasculature during liver resection. This marks the first implementation of real-time video segmentation of liver vasculature using this approach. Compared to traditional image-based segmentation models and other temporal models like FPN + EfficientNetV2-L, SgeFormer, ConvLSTM-U-Net and ViViT, Vivim substantially improves segmentation accuracy by effectively integrating inter-frame temporal information through its selective state space mechanism. It demonstrates excellent performance in vascular branch recognition, handling complex scenarios such as hemorrhage interference and instrument occlusion, as well as real-time high-resolution video segmentation. The system achieves a processing speed of up to 25 fps, providing immediate output without the need for image cropping, fully meeting intraoperative guidance requirements. In the multi-target recognition task, Vivim achieved a Dice score of 0.66 on the internal test set and 0.65 on the external test set, surpassing all other compared models. The incorporation of the boundary-sensitive HD95 validated Vivim’s superiority in capturing precise anatomical boundaries, while the inter-frame IoU (t‑IoU) and boundary jitter (b‑jitter) metrics provided a quantitative evaluation of its temporal consistency. Additionally, by incorporating metrics such as MAE and E-measure, the study further validated the comprehensive performance advantages of Vivim. Clinical validation indicates that Vivim effectively assists surgeons in improving the speed and accuracy of intraoperative vascular recognition, offering an innovative methodological framework for real-time liver surgery assistance. Its accuracy is comparable to that of novice surgeons but with the added capacity to identify lesions that might be overlooked by less experienced practitioners. This capability received high recognition during clinical testing, demonstrating its promise for meeting real-time decision-making demands in actual surgical environments. Vivim operates without the need for cropping or stitching preprocessing, enabling immediate input and output of video frames, which aligns well with intraoperative real-time application requirements^35^.

To thoroughly compare the performance of the Vivim model with other commonly used semantic segmentation models in clinical research, this study first summarizes and categorizes common surgical scenarios that may affect model recognition. Overall, video-based models demonstrated more precise segmentation compared to image-based models across various scenarios. For instance, the performance improvement from U-Net to its temporal extension (ConvLSTM-U-Net) and from SegFormer to SegFormer + T-Mamba was particularly notable, showing the substantial benefit of incorporating temporal modeling. Based on these categories, we conducted visualization and systematic comparison of model performance. In scenarios with surgical operation interference (Fig. 2) and hardware device interference (Fig. 3), our findings indicate that in clear, well-exposed fields (Fig. 2-I), Vivim precisely delineates blood vessels, closely matching GT. In more challenging scenes, such as bloodstained images (Fig. 2-II), small targets (Fig. 2-III), or simultaneous GP and HV presence (Fig. 2-IV), Vivim maintains superior robustness, while U-Net often fails in complex contexts, and all models exhibit difficulties distinguishing similar tissues (Fig. 2-V). Under hardware interference (Fig. 3), including instrument occlusion, smoke, low lighting, and blur, other models’ performance declines significantly; Vivim remains stable. Furthermore, as shown in Fig. 4, which displays three consecutive frames at 1-second intervals over a 3-second period, video-based models (particularly Vivim) demonstrated significantly improved segmentation consistency and accuracy compared to image-based models, clearly illustrating the advantage of leveraging temporal information. In clinical practice, Vivim’s occasional misclassifications minimally impact surgical judgments but effectively alert surgeons to exposed vessels, aiding complication prevention (Supplementary Table 6). For multi-target recognition (Task 2), Vivim distinguishes GP and HV reliably in favorable conditions (Fig. 5-I-III), but errors occur, such as confusing HV with GP or missing targets, often due to structural similarities and dataset limitations.

Critically, the temporal responsiveness analysis confirms that Vivim reacts to sudden anatomical changes with minimal delay (0.22 s TTFM) and without harmful oversmoothing, underscoring its clinical reliability (Supplementary Table 2). Specifically, when a vessel abruptly appears, Vivim only partially segments it, as the model requires time to accumulate sufficient spatiotemporal features from consecutive frames to confirm the complete contour and boundaries (Supplementary Fig. 1). This challenge is particularly pronounced for texture-ambiguous or low-contrast targets against the background, where single-frame information is insufficient. Additionally, small branches occupying few pixels are often blurred by motion, making them easily mistaken as noise or background in early frames. Conversely, when a vessel abruptly and partially disappears, Vivim does not promptly correct its prediction. This is likely due to ambiguous visual information near the occlusion boundary, which complicates determining the precise endpoint of the target and the onset of occlusion, resulting in fractured or missing predictions at boundaries. Moreover, as shown in Supplementary Fig. 1-IV, when a highly similar non-target structure appears, its visual features are too close to the target in feature space, confusing the classifier and causing Vivim to lose the target and segment the nearby similar structure instead. The observed under-smoothing during partial occlusions, however, highlights an area for future improvement.

We examined the model’s performance across diverse datasets and scenarios, noting that liver resection often involves distinguishing between branch and trunk vascular structures. To evaluate critical clinical tasks—such as transecting main trunk vessels—we created a test set excluding branch structures, focusing solely on main trunks, which significantly improved performance metrics like Dice score (Supplementary Table 3). This highlights the greater challenge of segmenting branches, likely due to their higher morphological variability and surrounding tissue interference. In the surgical workflow, however, both trunk and branch structures appear intermittently: branches during initial dissection, and the main trunk during transection, making a dedicated main-trunk-only model impractical, as frequent target switching would hinder workflow efficiency and increase misjudgment risk. A unified model capable of recognizing both structures provides better spatial consistency and clinical utility. Regarding vascular segmentation amidst tissue interference, cropping images to smaller regions (e.g., 800 × 800 pixels from 1920 × 1080) improved performance by reducing background noise, as shown in Supplementary Table 4. However, this approach introduces inference latency, as it requires overlapping patch division and stitching, and also introduces boundary artifacts, which complicate real-time application. Therefore, we favor simple resizing strategies to balance accuracy and system efficiency in clinical deployment.

Despite these advances, our study faces certain limitations. Firstly, though multi-center data were used in this study, the dataset is still relatively small. Given that data volume impacts deep learning performance, we’ll focus on building a larger multi-center dataset in the future. Secondly, while Vivim demonstrates superior performance on the external test set from Guangdong Second People’s Hospital, further validation across more diverse surgical centers is warranted to fully establish its generalizability. Thirdly, the prevalence of similar surgical scenes may lead to reduced model robustness; exploring new sampling strategies to enhance training data quality is necessary. Fourthly, although Vivim excels in single-label hepatic vasculature recognition, its capacity to differentiate between GP and HV remains limited, warranting further optimization. Fifthly, the irregular nature of vascular structures introduces challenges in annotation, as subjective labeling, such as unintentionally including hemorrhagic points or artifacts, can introduce bias and inconsistencies. Even if the model outputs anatomically precise boundaries, if GT annotations contain errors, traditional pixel-level metrics like Dice score may inappropriately penalize the model, failing to truly reflect its clinical utility. Hence, ensuring annotation quality is a crucial future consideration. Sixthly, a key priority is the development of uncertainty measure and visualization methods for the model’s predictions, which would provide crucial safety alerts in clinical deployment. Seventhly, the clinical deployment of our model is currently dependent on high-performance computing hardware (e.g., NVIDIA RTX 4090) to achieve real-time inference, which may limit its immediate accessibility in resource-constrained healthcare settings; future work will explore cloud-based deployment to broaden its applicability. Moreover, expanding the model’s recognition scope to achieve full-scene surgical understanding represents a critical evolution, encompassing the recognition and tracking of surgical instruments, detection and segmentation of intraoperative bleeding points, and identification of other critical anatomical landmarks. Most importantly, our long-term goal is to develop an intelligent surgical platform capable of full-scene perception. This platform will integrate functions such as identification of critical structures, instruments, and bleeding, and will be seamlessly integrated with navigation systems to provide intuitive visual guidance.

In conclusion, the Mamba-driven video segmentation AI model is highlighted for its novel application in recognizing key anatomical structures in LLR. It overcomes some limitations of image segmentation models by better leveraging surgical video information, efficiently balancing speed and precision.

## Methods

### Study design and participants

This retrospective, multicenter study received ethical approval from the Research Ethics Committee of each hospital: Zhujiang Hospital of Southern Medical University, the First Affiliated Hospital of Shihezi University, the First Affiliated Hospital of USTC, Tongji Hospital of Huazhong University of Science and Technology, and the Guangdong Second People’s Hospital. This study was conducted according to the tenets of the Declaration of Helsinki and was approved by Institutional Review Board (Trial registration: NCT06864702). As the study was retrospective in nature and images used were fully anonymized, the requirement for informed consent was waived. All experimental procedures were strictly conducted in accordance with relevant ethical guidelines and data protection regulations.

Between January 2021 and April 2024, 57 intraoperative videos (19402 images) of laparoscopic left lateral sectionectomy, left hepatectomy, and right hepatectomy were obtained from the five hospitals listed above. All surgeries were conducted for indications including colorectal liver metastases, hepatocellular carcinoma, and intrahepatic cholangiocarcinoma. The videos were converted into MP4 format, preprocessed at a resolution of 1920 × 1080 pixels and 24 fps. To enhance the model’s generalizability across different patients, the dataset comprised videos from 57 individual patients.

### Dataset

**Fig. 6:**
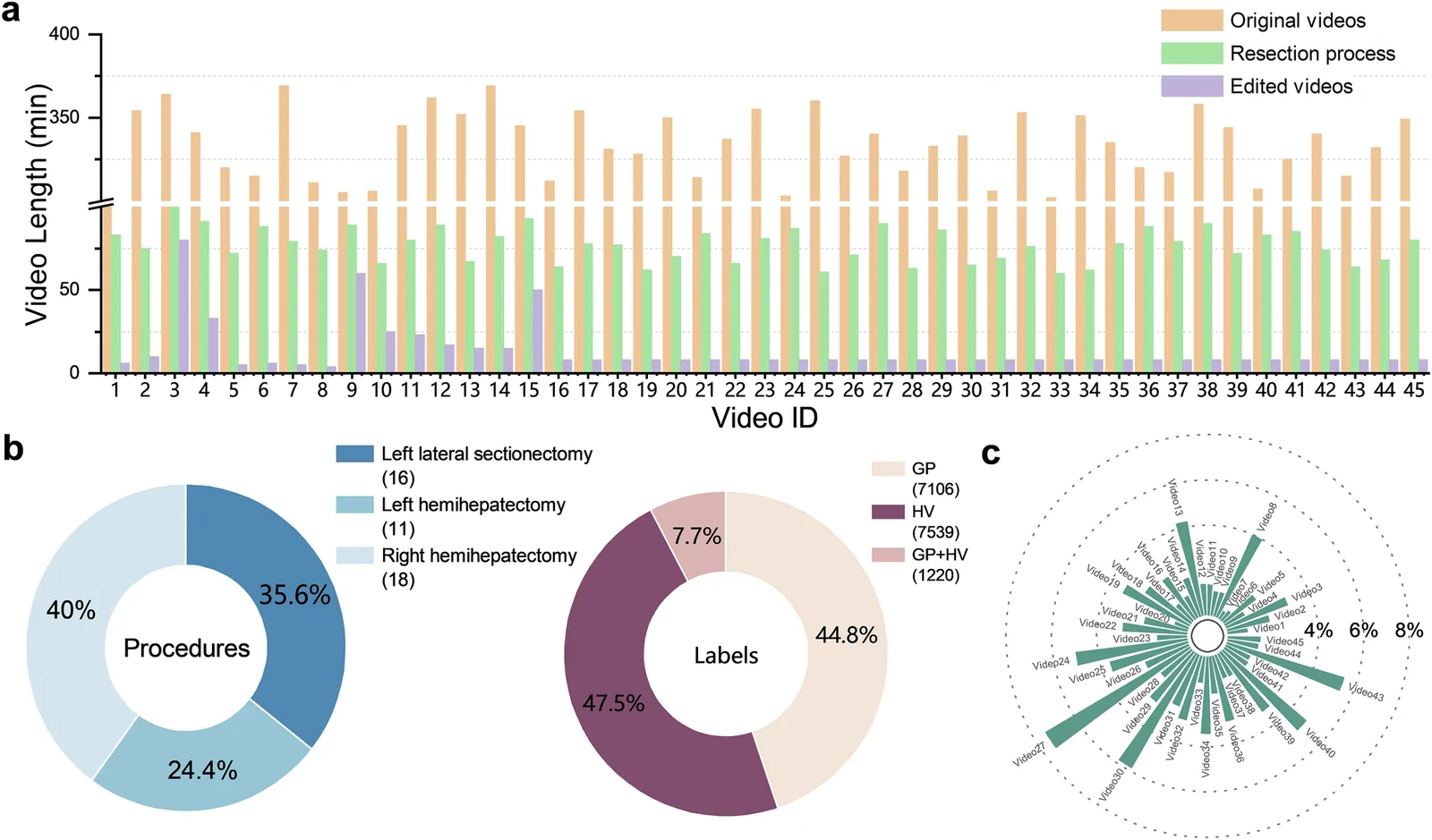
Videos and frames in the training and internal test dataset. **a** Lengths of all videos. The original videos contain clips of all the steps of laparoscopic surgery (from trocar insertion to abdominal incision closure. The resection process includes clips of all surgical steps for liver resection (from the beginning of the liver cutting surface exposure to the complete resection of the liver). The edited videos focus on clips of intrahepatic vascular exposure controlled within a specific duration. **b** The quantity ratio of different procedures and the proportion of labels contained in different video frames. **c** The average area ratio of the labeled area of each video to the total image area.

The dataset comprised a total of 19402 frames extracted from 57 surgical videos across five centers. From four of these centers, 45 videos (15865 frames) were utilized for model development. This subset was partitioned at a patient ratio of 7:3, resulting in a training set (33 videos, 12101 frames) and an internal test set (12 videos, 3764 frames). Patient and dataset characteristics of the training and internal test dataset are displayed in Supplementary Table 9. There were 29 males and 16 females in 45 patients. The mean age was 54.2 ( ± 6.5) years, and the mean Body Mass Index (BMI) was 21.6 ( ± 4.1) kg/m2. The lengths of the original, resection process, and edited videos in the training and internal test dataset are displayed in Fig. 6a. The average lengths of the original videos, resection process, and edited videos were 333.6 ( ± 20.3) min, 76.9 ( ± 10.3) min, and 13.2 ( ± 14.9) min, respectively. As shown in Fig. 6b, the dataset comprised 45 videos covering three surgical procedures: left lateral sectionectomy (n = 16), left hemihepatectomy (n = 11), and right hemihepatectomy (n = 18). A total of 15,865 video frames were annotated with two anatomical structures: GP alone (7,106 frames), HV alone (7,539 frames), and both GP and HV (1,220 frames). To quantify segmentation difficulty, we calculated the relative area of annotated targets within the entire image (Fig. 6c). The average target area in our dataset was 2.3% (±1.5%), significantly smaller than the 8.9% (±11.2%) reported for the video thyroid ultrasound segmentation dataset (VTUS), indicating higher complexity in our task due to smaller anatomical targets and increased background interference.

Additionally, a separate validation set of 5 videos (1378 frames) from Zhujiang Hospital was prepared for hyperparameter tuning. Furthermore, an independent external test set consisting of 7 videos (2159 frames) was sourced from the Guangdong Second People’s Hospital to rigorously assess model generalization. For the multi-target recognition task involving both GP and HV, we reprocessed the data to include labels with background, GP, and HV, mimicking the dataset scale of Task 1. For the multi-target recognition task involving both GP and HV, we reprocessed the data to include labels with background, GP, and HV, mimicking the dataset scale of Task 1.

### Data annotation

To reduce annotation workload, we selected key procedural segments from full surgical videos, starting from the exposure of the liver resection plane to the complete dissection of the hepatic hilum and hepatic veins. Due to minimal scene variation during surgery, frames were sampled at 1 fps, resulting in a total of 19,402 images. Annotations were performed on clearly visible GP and HV, excluding areas completely obscured by blood, instruments, or smoke, as these structures are difficult to observe reliably regardless of the surgeon’s experience, and annotation quality cannot be guaranteed.

Annotation was carried out using LabelMe (MIT, Boston), version 3.16, compatible with Windows 10 or higher. To address the burden caused by the large volume of dataset, a three-tier quality control system was established to ensure the accuracy and reliability of data annotation. Our annotation team consisted of 8 hepatobiliary-pancreatic (HBP) surgeons, including 4 skilled surgeons (>10 laparoscopic hepatectomy cases in 2 years of surgical experience), who annotate the GP and HV in laparoscopic images; 2 intermediate-level surgeons (>50 laparoscopic hepatectomy cases in 5 years of surgical experience), who conducted preliminary quality control; 2 senior HBP surgeons (>200 laparoscopic hepatectomy cases) served as auditors responsible for quality control of the annotated images. To ensure annotation quality, the process followed strict protocols. First, two senior surgeons established annotation guidelines through discussion. Annotators received at least 3 h of training by senior surgeons on anatomical recognition and annotation rules. Next, four primary surgeons independently annotated data from 1–2 patients each. During annotation, any issues were immediately communicated to intermediate-level surgeons, who could give real-time advice and adjust strategies. After initial annotation, the intermediate-level surgeons reviewed all frames for their assigned data, identified and corrected any inaccuracies, then confirmed the revisions. Finally, senior surgeons performed an overall review. If discrepancies remained, corrections were made, and the review cycle repeated until standards were met. Only annotations passing all quality controls were used as standard references.

### Model performance evaluation metrics and model training

The overall experimental workflow is schematically illustrated in Supplementary Fig. 2. Our study encompassed a comprehensive comparison of seven distinct models to ensure a robust evaluation. These included both image-based and video-based architectures. The image-based models consisted of U-Net^26^, DeepLab v3 + ^27^, nnU-Net^28^, FPN + EfficientNetV2-L and SegFormer^29–31^. The video-based models comprised ConvLSTM-U-Net, ViViT^32^, SegFormer + T-Mamba, and Vivim^23^. All models were implemented using publicly available source codes. During model development, we individually optimized the hyperparameters for each model on the validation set rather than employing a fixed configuration, ensuring a fair comparison and enabling each model to achieve its optimal performance on our dataset. Model configurations and training protocols are shown in Supplementary Table 10. The segmentation outputs generated by these networks were compared with the manually annotated GT. Performance metrics including Dice score, F1 score, Intersection over Union (IoU), precision, recall, Mean Absolute Error (MAE), E-Measure^36^, HD95, t-IoU, and b-jitter were used to quantitatively assess the segmentation algorithms^23,34^. In model evaluation, a Dice score of 1 indicates perfect segmentation overlap, whereas 0 indicates no overlap between the predicted and manual annotations. The pixel-wise binary classification task results are characterized by four outcomes: True Positive (TP, predicted as positive and GT is positive), True Negative (TN, predicted negative and GT is negative), False Negative (FN, predicted negative but GT is positive), and False Positive (FP, predicted positive but GT is negative). The detailed formulas for each metric applied to pixel-level classification are illustrated in Supplementary Fig. 3.

To evaluate the models’ segmentation performance across different anatomical structures, data integration was performed by combining GP and HV labels. Two modes of training data were used: a single-target recognition mode where GP and HV were merged into a single label (referred to as Task 1: GP or HV), and a multi-target recognition mode where GP and HV were distinguished as separate labels (referred to as Task 2: GP + HV). All models were trained on the dataset provided in this study, with adjustments and refinements made during fine-tuning to optimize for these tasks.

The training was performed on an Ubuntu 20.04 platform equipped with a GeForce RTX 4090 GPU and 24 AMD EPYC 7302 16-Core Processors.

## Supplementary information

Supplementary Information

Supplementary Movie 1

## Data Availability

The datasets generated and analyzed during the current study are not publicly available due to ethical regulations on confidentiality and privacy but are available from the corresponding author on reasonable request.

## Code availability

The AI models developed in this study include U-Net, DeepLab v3 + , nnU-Net, FPN + EfficientNetV2-L, SegFormer, ConvLSTM-U-Net, ViViT, SegFormer + T-Mamba, and Vivim. The underlying codes for all models are available on GitHub. The links are as follows: U-Net is hosted at https://github.com/milesial/Pytorch-UNet/blob/master/unet/unet_model.py; DeepLab v3+ is hosted at https://github.com/bubbliiiing/deeplabv3-plus-pytorch/blob/main/nets/deeplabv3_plus.py; nnU-Net is hosted at https://github.com/MIC-DKFZ/nnUNet;SegFormer is hosted at https://github.com/NVlabs/SegFormer; FPN + EfficientNetV2-L, ConvLSTM-U-Net and SegFormer + T-Mamba are hosted at https://github.com/Yaostars/UNet_LSTM-EfficientNet_FPN; ViViT is hosted at https://github.com/google-research/scenic/tree/main/scenic/projects/vivit; Vivim is hosted at: https://github.com/scott-yjyang/Vivim.
