# Supplementary material for: Real-time anatomy recognition in laparoscopic liver resection using video segmentation AI model
Source: NPJ Digit Med. 2026 Jul 10;9:838. doi: 10.1038/s41746-026-02989-5 (PMC13728136; doi:10.1038/s41746-026-02989-5)
Supplement: Supplementary file 1 [file 41746_2026_2989_MOESM1_ESM.pdf]

Real-time Anatomy Recognition in Laparoscopic Liver Resection Using Video Segmentation AI Model

SUPPLEMENTARY NOTES

1.Clinical performance evaluation *pag. 2*

Tables and Figures

Supplementary Table 1. *pag. 3*

Supplementary Table 2 *pag. 4*

Supplementary Table 3 *pag. 5*

Supplementary Table 4 *pag. 6*

Supplementary Table 5 *pag. 7*

Supplementary Table 6 *pag. 8*

Supplementary Table 7 *pag. 9*

Supplementary Table 8 *pag.10*

Supplementary Table 9 *pag.11*

Supplementary Table 10 *pag.12*

Supplementary Figure 1 *pag.13*

Supplementary Figure 2 *pag. 14*

Supplementary Figure 3 *pag. 15*

Supplementary Movie 1 *pag. 16*

SUPPLEMENTARY NOTES

Supplementary Note 1: Clinical performance evaluation

We compared the speeds at which surgeons and the AI model Vivim recognised and visualised data from multiple perspectives to evaluate whether the AI model could accurately recognise anatomical landmarks (GP or HV). We investigated whether the AI model's recognition performance was equal to or higher than that of the primary surgeons. Several measures were implemented to minimise system errors: 1. To reduce reaction time, various methods were tested, with participants placing their hands directly on the pause button being the fastest method for immediate intervention; 2. To further decrease the impact of delay, participating surgeons received training prior to testing to familiarise themselves with pausing the video by clicking. Repeated exercises significantly shortened response times, rendering delays negligible; 3. To reduce system bias, six primary surgeons participated in recognition assessments.

Supplementary Table 1:
Uncertainty Evaluation Results of Vivim on the Internal Test Set.

Level	Metric	Value
Pixel	Pearson (U, error)	0.440
	ECE (%)	1.15
	NLL	0.070
	Brier	0.014

U denotes pixel-level predictive entropy, *ECE* expected calibration error; *NLL* negative log-likelihood; *Brier* Brier score.

Supplementary Table 2
Quantitative analysis of temporal responsiveness and oversmoothing of Vivim on surgical video events from the internal test dataset.

Event Type	Number	Avg. Detection Lag (s)*	Avg. TTFM (s)†	Over-smoothing Failures‡	Under-smoothing Cases§
Abrupt Appearance	289	2.07	-	0	-
Abrupt Partial Disappearance	1539	4.14	0.22	0	1503

TTFM mean Time-to-First-Correct-Mask.

* Measures adaptation pace in the 1 fps sampled sequence. It is the frame index difference (seconds) between the annotated event frame and the first subsequent frame where the model's IoU meets the threshold (>0.7 for appearance; change >0.2 for disappearance). † Real-world response time in the original 24 fps video, normalized from the downsampled sequence. It reflects the speed of generating the first accurate clinical assessment. ‡ Occurs if, after a sharp drop in ground truth vessel area (partial disappearance), the model's prediction retains a high vessel ratio (in-event IoU >0.5 or post-event IoU >0.3) due to excessive temporal smoothing ("ghosting"). § Indicates temporal instability (frame-to-frame jitter) during an event, defined by a Local Mask Stability (LMS) score < 0.5. The high count highlights that maintaining stability under visual ambiguity is the primary challenge.

Supplementary Table 3:

Performance metrics of U-Net, DeepLab v3+, and Vivim for the recognition of vascular trunks, including a comparison of metrics for recognizing both branches and trunks together versus recognizing trunks alone.

Model	Task	Dice Score (F1 Score)	mIoU	Accuracy	Precision	Recall	MAE	E-Measure
U-Net	Branches + trunks	0.44 (0.30)	0.32 (0.25)	0.99 (0.01)	0.55 (0.35)	0.47 (0.34)	0.02	0.70
DeepLab v3+	Branches + trunks	0.46 (0.21)	0.32 (0.16)	0.97 (0.01)	0.48 (0.23)	0.54 (0.32)	0.04	0.71
Vivim	Branches + trunks	0.71 (0.25)	0.59 (0.25)	0.99 (0.01)	0.72 (0.27)	0.76 (0.26)	0.01	0.89
U-Net	trunks	0.46 (0.24)	0.35 (0.19)	0.99 (0.01)	0.62 (0.31)	0.49 (0.31)	0.02	0.70
DeepLab v3+	trunks	0.48 (0.17)	0.34 (0.13)	0.98 (0.01)	0.47 (0.18)	0.68 (0.24)	0.03	0.72
Vivim	trunks	0.75 (0.22)	0.63 (0.17)	0.99 (0.01)	0.77 (0.23)	0.81 (0.21)	0.01	0.90

Data were expressed in mean (±standard deviation, SD). In this study, the Dice score and F1 score have the same values, and recall is also referred to as sensitivity. *mIoU* mean intersection over union, *MAE* mean absolute error, *Vivim* Video Vision Mamba-based framework.

Supplementary Table 4:

Performance metrics of U-Net, DeepLab v3+, and Vivim for Task 1 (GP or HV), trained and tested on a dataset cropped to a resolution of 800×800, along with a comparison of metrics before and after cropping.

Model	Operation	Dice Score (F1 Score)	mIoU	Accuracy	Precision	Recall	MAE	E-Measure
U-Net	before cropping	0.44 (0.30)	0.32 (0.25)	0.99 (0.01)	0.55(0.35)	0.47(0.34)	0.02	0.70
DeepLab v3+	before cropping	0.46 (0.21)	0.32 (0.16)	0.97 (0.01)	0.48(0.23)	0.54(0.32)	0.04	0.71
Vivim	before cropping	0.71 (0.25)	0.59 (0.25)	0.99 (0.01)	0.72(0.27)	0.76(0.26)	0.01	0.89
U-Net	after cropping	0.52(0.27)	0.39(0.23)	0.98(0.01)	0.65(0.29)	0.51(0.31)	0.06	0.72
DeepLab v3+	after cropping	0.53(0.19)	0.40(0.17)	0.96(0.01)	0.57(0.21)	0.58(0.28)	0.07	0.69
Vivim	after cropping	0.74(0.21)	0.63(0.22)	0.98(0.01)	0.76(0.24)	0.78(0.23)	0.03	0.88

Data were expressed in mean (±standard deviation, SD). In this study, the Dice score and F1 score have the same values, and recall is also referred to as sensitivity. *mIoU* mean intersection over union, *MAE* mean absolute error.

Supplementary Table 5

Performance metrics for Task 1 (GP or HV) using image-based and video-based models on the DSAD.

Model	Type	Dice Score (F1 Score)	mIoU	Accuracy	Precision	Recall	MAE	E-Measure	HD95	t-IoU	b-jitter
U-Net	Image	0.44 (0.26)	0.32 (0.21)	0.98 (0.02)	0.44 (0.27)	0.54 (0.33)	0.04	0.76 (0.15)	136.61 (7.55)	0.35 (0.05)	37.84 (2.98)
DeepLab v3+	Image	0.50 (0.23)	0.37 (0.20)	0.98 (0.01)	0.59 (0.28)	0.52 (0.28)	0.02	0.79 (0.15)	91.18 (6.90)	0.39 (0.03)	40.85 (2.16)
nnU-Net	Image	0.40 (0.23)	0.27 (0.19)	0.97 (0.02)	0.39 (0.27)	0.52 (0.29)	0.10	0.67 (0.12)	156.33 (7.31)	0.29 (0.03)	31.24 (1.74)
FPN + EfficientNetV2-L	Image	0.42 (0.26)	0.30 (0.21)	0.98 (0.02)	0.48 (0.29)	0.47 (0.31)	0.03	0.74 (0.14)	116.04 (7.11)	0.32 (0.03)	39.62 (2.78)
SegFormer	Image	0.58 (0.27)	0.44 (0.21)	0.98 (0.01)	0.56 (0.25)	0.70 (0.27)	0.02	0.80 (0.14)	91.36 (7.63)	0.47 (0.03)	38.31 (2.61)
ConvLSTM-U-Net	Video	0.42 (0.26)	0.30 (0.22)	0.98 (0.02)	0.45 (0.27)	0.49 (0.34)	0.03	0.76 (0.18)	133.32 (7.98)	0.34 (0.08)	36.51 (0.49)
ViViT	Video	0.37 (0.26)	0.26 (0.21)	0.98 (0.02)	0.43 (0.31)	0.42 (0.33)	0.03	0.70 (0.18)	136.58 (9.21)	0.31 (0.08)	49.81 (0.39)
SegFormer + T-Mamba	Video	0.58 (0.24)	0.44 (0.22)	0.98 (0.02)	0.51 (0.26)	0.76 (0.26)	0.02	0.80 (0.16)	104.10 (8.68)	0.48 (0.05)	30.47 (1.74)
Vivim	Video	0.59 (0.25)	0.45 (0.23)	0.98 (0.02)	0.62 (0.27)	0.63 (0.29)	0.02	0.84 (0.13)	80.94 (7.57)	0.49 (0.06)	36.21 (2.40)

Data were expressed in mean (±standard deviation, SD). In this study, the Dice score and F1 score have the same values, and recall is also referred to as sensitivity. mIoU mean intersection over union, MAE mean absolute error, t-IoU Inter-frame IoU, b-jitter boundary jitter.

Supplementary Table 6:
Comparison of recognition performance of primary surgeons and the Vivim model.

Target task	Primary surgeon-first recognition, No. (%)	Vivim-first recognition, No. (%)	Simultaneous recognition, No. (%)	Vivim recognition, No. (%)
Task 1				
GP or HV	2 (10.0)	18 (90.0)	0 (0)	19 (95.0)
GP	1	6	0	16
HV	2	13	0	18
Task 2				
GP + HV	5 (38.5)	8 (61.5)	0 (0)	10 (50.0)
GP	3	2	0	6
HV	5	7	0	9

Supplementary Table 7:
Likert scale.

Aim	Questions				
Segmentation accuracy	1. The anatomical targets segmented by the model closely match the actual targets.				
Preservation of segmentation details	2. The model is capable of clearly identifying small anatomical structures.				
Consistency of segmentation results	3. The model's anatomical segmentation results remain consistent across different video segments.				
Interpretability of segmentation results	4. I can easily understand the anatomical segmentation results provided by the model.				
Quality of segmentation result visualization	5. The anatomical segmentation images provided by this model are of high quality, making them easy to view and analyze.				
Segmentation efficiency	6. The model performs segmentation in liver resection videos quickly enough to meet clinical demands.				
Not satisfied at all 1	2	3	4	5	Very satisfied

Supplementary Table 8:
Comparison of segmentation performance ratings for Task 1 between novice and expert surgeons using a Likert scale.

Questions	Novice Group Avg (n=6)	Expert Group Avg (n=7)	Overall Avg (N=13)
1. The anatomical targets segmented by the model closely match the actual targets.	4.50	3.86	4.15
2. The model is capable of clearly identifying small anatomical structures.	4.17	3.43	3.77
3. The model's anatomical segmentation results remain consistent across different video segments.	4.50	3.71	4.08
4. I can easily understand the anatomical segmentation results provided by the model.	4.67	3.86	4.23
5. The anatomical segmentation images provided by this model are of high quality, making them easy to view and analyze.	4.67	3.86	4.23
6. The model performs segmentation in liver resection videos quickly enough to meet clinical demands.	4.50	3.57	4.00

Supplementary Table 9:
Patient characteristics and lesion distribution according to metastatic extents and locations in the whole dataset.

Characteristics	Value
Number of Patients	45
Age, mean, years	54.2
BMI, mean, kg/m^2	21.6
Sex	
Male	29
Female	16
Process	
Left lateral sectionectomy	16
Left hemihepatectomy	11
Right hemihepatectomy	18
Number of Images	
Task 1 (GP or HV)	15865
Task 2 (GP + HV)	15865
Pathological diagnosis	
Colorectal liver metastases	5
Hepatocellular carcinoma	32
Intrahepatic cholangiocellular	8

Supplementary Table10:
Model Configurations and Training Protocols.

Model	Type	Input Resolution	Learning Rate	Optimizer	Weight Decay	LR Scheduler	Batch Size (Clips × Frames)	Epochs	Loss Function
U-Net	Image	512×512	0.0001	AdamW	0.0001	CosineAnnealing	16 (images)	100	$\mathcal{L}_{\text{Dice}} + \mathcal{L}_{\text{CE}}$
DeepLab v3+	Image	512×512	0.0002	AdamW	0.0005	CosineAnnealing	16 (images)	100	$\mathcal{L}_{\text{Dice}} + \mathcal{L}_{\text{CE}}$
nnU-Net	Image	512×512	0.01	SGD (mom.=0.99)	0.00003	PolyLR	16 (images)	200	$\mathcal{L}_{\text{Dice}} + \mathcal{L}_{\text{CE}}$
FPN+EfficientNetV2-L	Image	512×512	0.0001	AdamW	0.0001	CosineAnnealing	16 (images)	100	$\mathcal{L}_{\text{Dice}} + \mathcal{L}_{\text{CE}}$
SegFormer	Image	512×512	0.00006	AdamW	0.01	CosineAnnealing	16 (images)	100	$\mathcal{L}_{\text{Dice}} + \mathcal{L}_{\text{CE}}$
ConvLSTM-U-Net	Video	512×512	0.0001	AdamW	0.0001	CosineAnnealing	2 clips × 5	100	$\mathcal{L}_{\text{Dice}} + \mathcal{L}_{\text{CE}}$
ViViT	Video	512×512	0.00005	AdamW	0.05	CosineAnnealing	2 clips × 5	100	$\mathcal{L}_{\text{Dice}} + \mathcal{L}_{\text{CE}}$
SegFormer + T-Mamba	Video	512×512	0.00006	AdamW	0.01	CosineAnnealing	2 clips × 5	100	$\mathcal{L}_{\text{Dice}} + \mathcal{L}_{\text{CE}}$
Vivim	Video	512×512	0.00002	AdamW	0.01	CosineAnnealing	2 clips × 5	100	$\mathcal{L}_{\text{seg}} + \lambda_1\mathcal{L}_{\text{affine}} + \lambda_2\mathcal{L}_{\text{bce}}$

All models were trained using the same pre-processing (resize to 512×512, same augmentations) and the same hardware (NVIDIA RTX 4090). The optimal hyperparameters for each model were selected based on performance on the independent validation set.

Supplementary Figure 1. Failure cases of Vivim segmentation related to temporal modeling latency and over-smoothing in dynamic surgical scenes. I–II delayed mask update after instrument retraction; **III–IV** persistent false segmentation after occlusion. GT, ground truth; Pred, prediction**.**

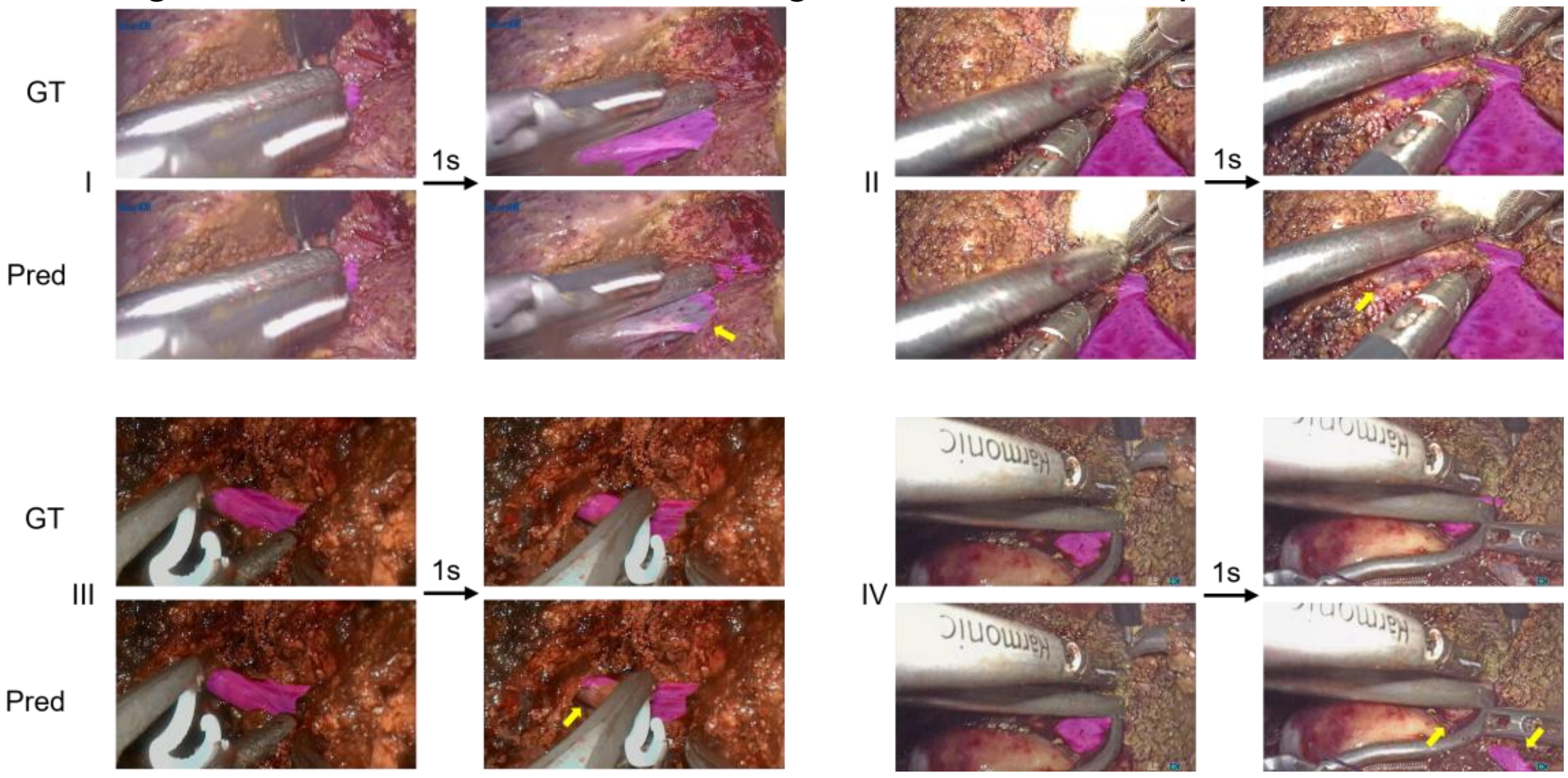

Supplementary Figure 2. Schematic of the model development, validation, and evaluation workflow.

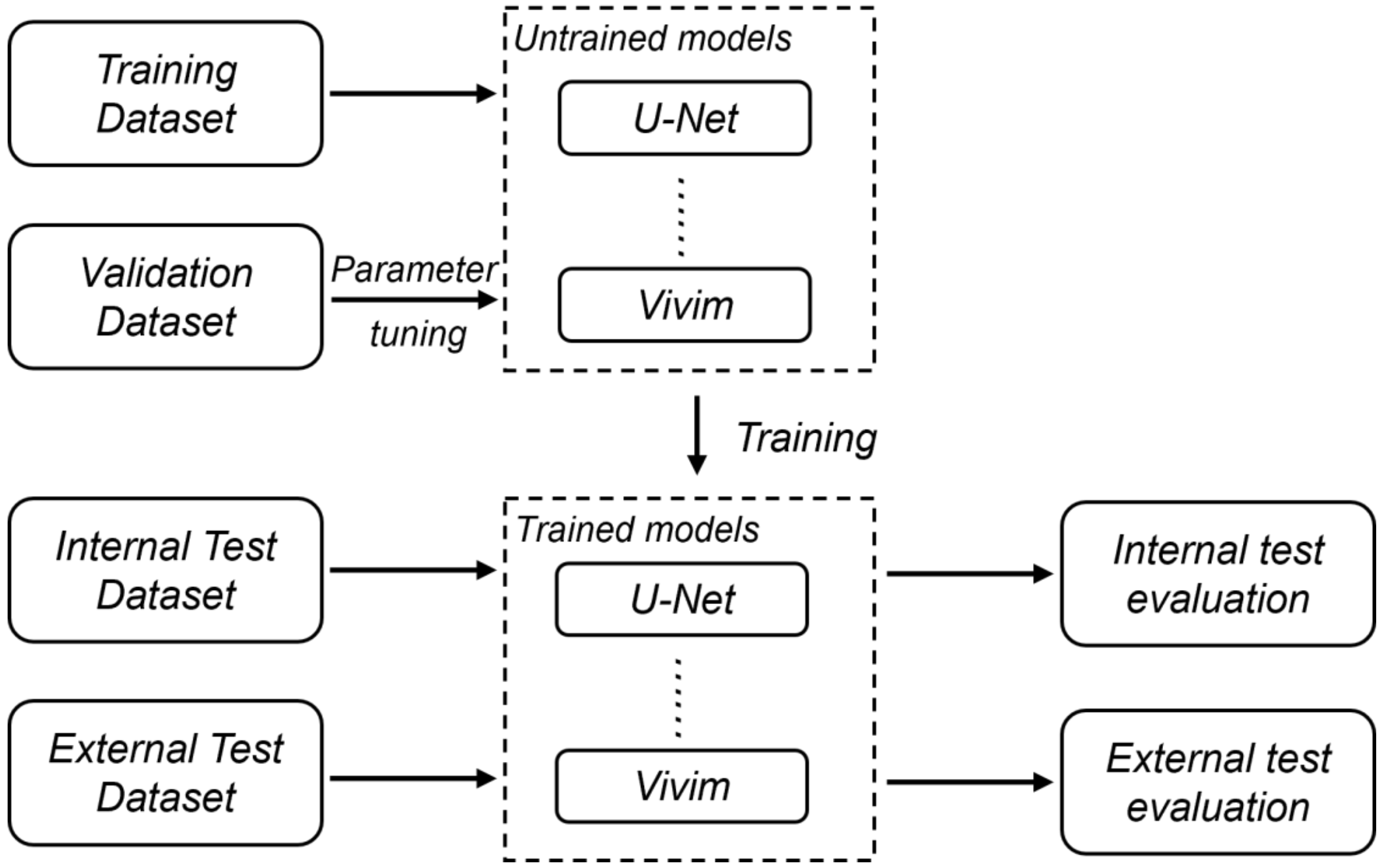

Supplementary Figure 3. Illustration and calculation formulas for performance metrics of AI models.

a

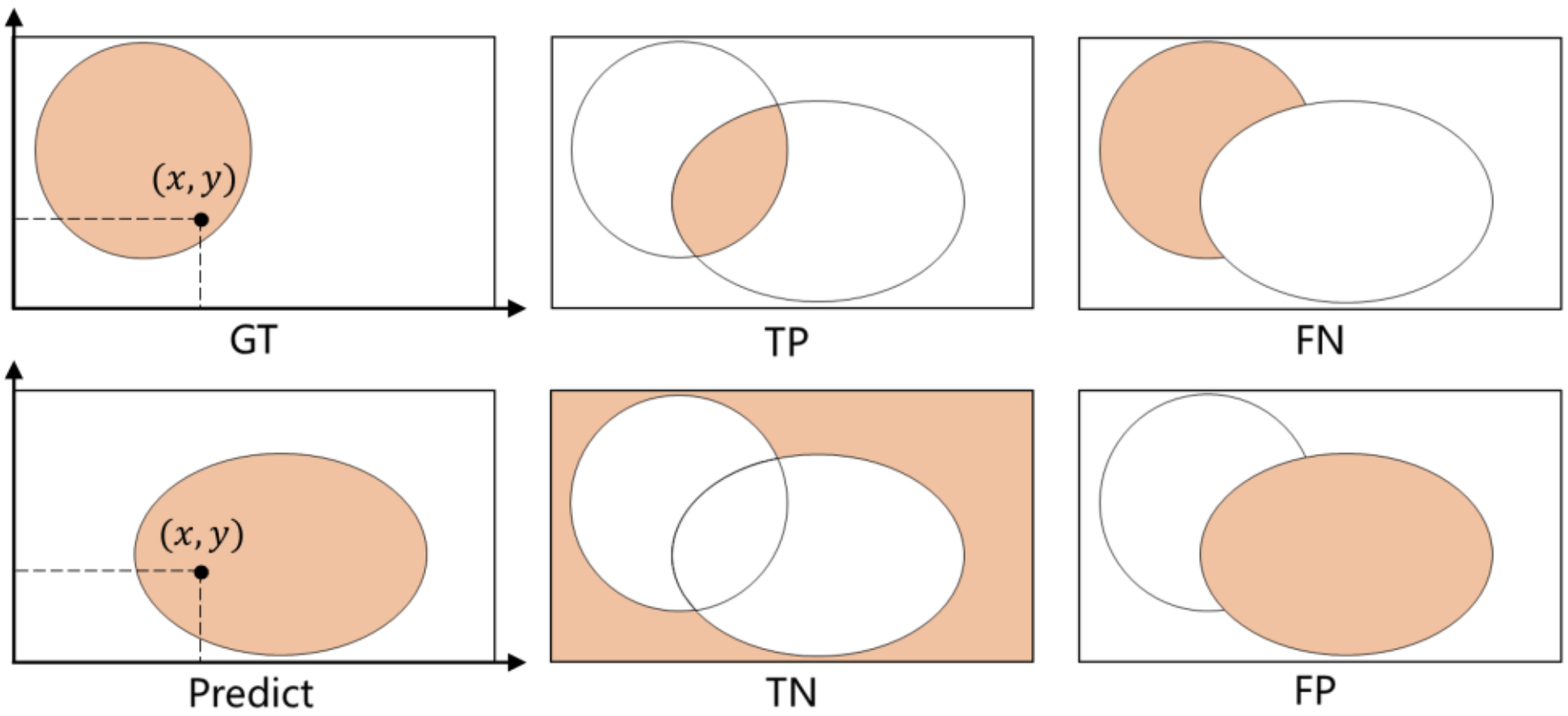

b

**A** $\text{Dice Score} = \frac{2 \times TP}{GT + Predict}$	**B** $\text{IoU} = \frac{TP}{TP + FN + FP}$	**C** $\text{F1 Score} = \frac{2 \times TP}{2 \times TP + FN + FP}$
**D** $\text{Accuracy} = \frac{TP + TN}{TP + TN + FN + FP}$	**E** $\text{Precision} = \frac{TP}{TP + FP}$	**F** $\text{Recall} = \frac{TP}{TP + FN}$
**G** $\text{MAE} = \frac{\lvert GT - Predict \rvert}{TP + TN + FN + FP}$	**H** $E = \frac{1}{w \times h} \sum_{x=1}^{w} \sum_{y=1}^{h} \frac{1}{4} \left(1 + \frac{2(GT(x,y) - \mu_{GT})(Predict(x,y) - \mu_{Predict})}{(GT(x,y) - \mu_{GT})^2 + (Predict(x,y) - \mu_{Predict})^2}\right)^2$	

a shows illustration of ground truth (GT), true positive (TP), false negative (FN), predicted positive (Predict), true negative (TN), and false positive (FP). **b** shows calculation formula of different metrics. **(A)** calculation formula for the Dice score. **(B)** calculation formula for the Intersection over Union (IoU). **(C)** calculation formula for the F1 score. **(D)** calculation formula for accuracy. **(E)** calculation formula for precision. **(F)** calculation formula for recall. **(I)** calculation formula for mean absolute error (MAE). **(H)** calculation formula for the E-measure.

Supplementary Movie 1. Real-time identification of intraoperative target structures.
